# Tetramethoxystilbene-Loaded Liposomes Restore Reactive-Oxygen-Species-Mediated Attenuation of Dilator Responses in Rat Aortic Vessels Ex vivo

**DOI:** 10.3390/molecules24234360

**Published:** 2019-11-29

**Authors:** Azziza Zaabalawi, Cai Astley, Lewis Renshall, Frances Beards, Adam P. Lightfoot, Hans Degens, Debra Whitehead, Yvonne Alexander, Lynda K Harris, May Azzawi

**Affiliations:** 1 Centre for Bioscience, Faculty of Science and Engineering, Manchester Metropolitan University, Chester Street, Manchester M1 5GD, UK; azziza.z.zaabalawi@stu.mmu.ac.uk (A.Z.); cai.astley@stu.mmu.ac.uk (C.A.); Y.Alexander@mmu.ac.uk (Y.A.); 2 Division of Pharmacy and Optometry, University of Manchester, Oxford Road, Manchester M13 9PL, UK; lewis.renshall@manchester.ac.uk (L.R.); frances.beards@manchester.ac.uk (F.B.); Lynda.K.Harris@manchester.ac.uk (L.K.H.); 3 Maternal and Fetal Health Research Centre, Faculty of Biology, Medicine and Health, University of Manchester, Oxford Road, Manchester M13 9WL, UK; 4 Maternal and Fetal Health Research Centre, Manchester University NHS Foundation Trust, Manchester Academic Health Sciences Centre, St Mary’s Hospital, Manchester M13 9WL, UK; 5 Centre for Musculoskeletal Science and Sports Medicine, Faculty of Science and Engineering, Manchester Metropolitan University, Chester Street, Manchester M1 5GD, UK; A.Lightfoot@mmu.ac.uk (A.P.L.); H.Degens@mmu.ac.uk (H.D.); 6 Institute of Sport Science and Innovations, Lithuanian Sports University, LT-44221 Kaunas, Lithuania; 7 Advances Materials and Surface Engineering Research Centre, Faculty of Science and Engineering, Manchester Metropolitan University, Chester Street, Manchester M1 5GD, UK; D.Whitehead@mmu.ac.uk

**Keywords:** liposomes, 2,3′,4,5′-tetramethoxystilbene, endothelium, aorta, oxidative stress, reactive oxygen species, nitric oxide, vascular function

## Abstract

The methylated analogue of the polyphenol resveratrol (RV), 2,3′,4,5′-tetramethoxystilbene (TMS) displays potent antioxidant properties and is an effective cytochrome P450 (CYP) 1B1 inhibitor. The bioavailability of TMS is low. Therefore, the use of liposomes for the encapsulation of TMS is a promising delivery modality for enhanced uptake into tissues. We examined the effect of delivery of TMS in liposomes on the restoration of vasodilator responses of isolated aortic vessels after acute tension elevation ex vivo. Aortic vessels from young male Wistar rats were isolated, and endothelial-dependent (acetylcholine, ACh) and -independent (sodium nitroprusside, SNP) responses assessed. Acute tension elevation (1 h) significantly reduced ACh dilator responses, which were restored following incubation with superoxide dismutase or apocynin (an NADPH oxidase inhibitor). Incubation with TMS-loaded liposomes (mean diameter 157 ± 6 nm; PDI 0.097) significantly improved the attenuated dilator responses following tension elevation, which was sustained over a longer period (4 h) when compared to TMS solution. Endothelial denudation or co-incubation with L-NNA (Nω-nitro-l-arginine; nitric oxide synthase inhibitor) resulted in loss of dilator function. Our findings suggest that TMS-loaded liposomes can restore attenuated endothelial-dependent dilator responses induced by an oxidative environment by reducing NADPH-oxidase-derived ROS and potentiating the release of the vasodilator nitric oxide. TMS-loaded liposomes may be a promising therapeutic strategy to restore vasodilator function in vascular disease.

## 1. Introduction

The vascular endothelial lining of blood vessels plays a pivotal role in the synthesis and release of highly regulated vasoactive substances, including the potent vasodilator nitric oxide (NO). An imbalance in the release of endothelial-derived vasodilator and vasoconstrictor mediators causes endothelial dysfunction (ED), which results in a dominant pro-vasoconstrictor state due to the generation of reactive oxygen species (ROS) and a consequent reduction in NO bioavailability [1,2]. ED is also associated with increased constriction, partly attributed to the decline in NO-mediated inhibition of potent vasoconstrictors such as 20-hydroxyeicosatetraenoic acid (20 HETE). The latter is a cytochrome P450 (CYP) 1B1 eicosanoid, which has been shown to contribute to the development of hypertension [3]. Attractive supplements to current treatment strategies include plant-derived polyphenols such as resveratrol (RV; 3,5,4′-trihydroxystilbene), due to its anti-oxidant properties and ability to regulate the endothelial nitric oxide synthase enzyme (eNOS) [4,5]. Recent studies have been dedicated to developing derivatives of RV that have greater bioavailability and pharmacological activity than RV [6]. In particular, the methylated derivative of RV 2,3′,4,5′-tetramethoxystilbene (TMS) has been demonstrated to be 1000 times more potent in inhibiting CYP1B1 than RV in spontaneously hypertensive rats (SHR) [7]. Elevated CYP1B1 activity, endothelial dysfunction, ROS generation, NADPH oxidase activity, and expression of *NOX-1*, *ERK1/2*, and *p38 MAPK* were diminished by TMS in the aorta, heart, and kidney of the SHR model [7,8]. Intravenous administration of TMS in rats showed moderate clearance (46.5 ± 7.6 mL/min/kg) [9] and better bioavailability (4.5%) [10], compared to RV; however, the absolute oral bioavailability of TMS remained low (6.31 ± 3.30%) [9].

Liposomes are recognized as promising agents for drug delivery due to their biocompatibility and ability to cross physiological barriers [11,12]. Liposomes have been clinically approved for various medical applications including anticancer treatment, owing to their high therapeutic efficacy and low cytotoxicity. Major progress was made in their clinical application with the development of PEGylated liposomes. Displaying PEG (*N*-[amino (polyethylene glycol)] on the liposomal surface increases their circulation time by preventing opsonisation, uptake, and elimination by phagocytes [12]. TMS encapsulated in PEGylated liposomes could thus serve as an attractive delivery system while preserving the stability of TMS and enhancing its bioavailability [13]. The aim of this study was to assess the potential of TMS-loaded liposomes to restore the reduced vasodilator capacity of isolated aortic vessels exposed to acute tension elevation ex vivo.

## 2. Results

### 2.1. Production and Characterisation of TMS-Loaded Liposomes

Liposomes (blank liposomes) with a mean diameter of 182 ± 3 nm, polydispersity index (PDI) of 0.081, and an average zeta potential of −13.17 ± 0.76 mV were dispersed in phosphate-buffered saline (PBS). TMS-loaded liposomes had a mean diameter of 157 ± 6 nm, PDI of 0.097, and an average zeta potential of −13.13 ± 0.67 mV, whereas liposomes containing the dye, 5(6)-carboxyfluorescein, and TMS had a mean diameter of 150 ± 3 nm, PDI of 0.070, and an average zeta potential of −15.80 ± 1.31 mV. All measurements were conducted at pH 7.2–7.4.

**Figure 1:**
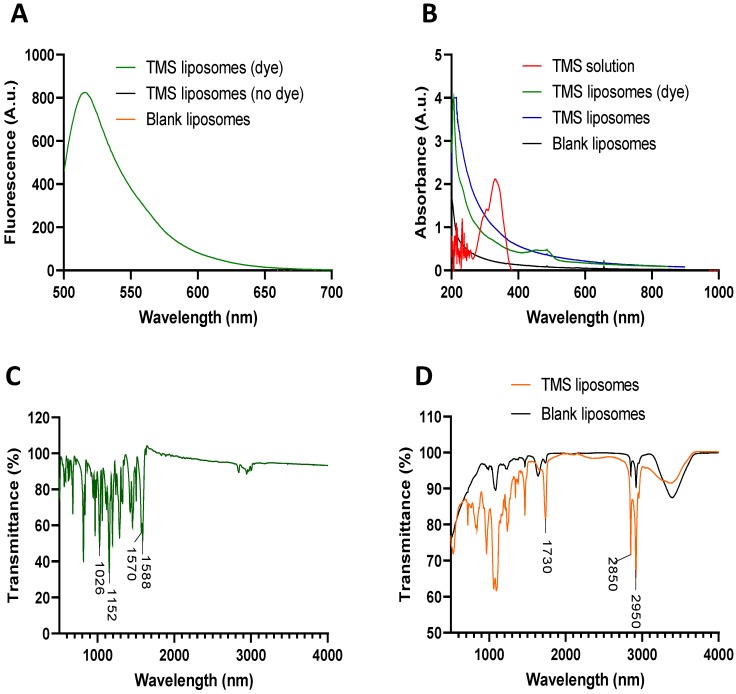
Chemical characterization of tetramethoxystilbene (TMS) and TMS-loaded liposomes. Fluorescence spectroscopy displaying spectra between 500–700 nm (**A**). Ultraviolet–visible spectroscopy (UV-Vis) displaying absorbance spectra between 200–1000 nm (**B**). Fourier-transform infrared spectroscopy (FTIR) profiles of TMS powder (**C**) and TMS-loaded liposomes (**D**), displaying transmittance (%) ranging between 500 and 4000 nm.

Structural and functional characterisation of PEGylated liposomes was established using chemical techniques to confirm dye and drug entrapment within the liposomes. Fluorescence peaks were detected at 517 nm (corresponding to the dye) from TMS–dye-encapsulated, but not blank or TMS-loaded liposomes (Figure 1A). Independent analysis of TMS solution identified two absorbance peaks at 302 and 326 nm; however, no peaks corresponding to TMS were distinguishable in either TMS-or TMS–dye-encapsulated liposomes (Figure 1B). TMS has a similar infra-red profile to its well-characterized parent compound RV, with characteristic benzene valence v(C=C) vibrations identifiable at 1588 and 1570 cm^−1^. The strong, broad band characteristics of trans-RV were noticeably absent between 3550–3200 cm^−1^; however, several bands were identifiable at 2850, 2952, and 3000 cm^−1^, corresponding to the valence vibration and stretching of O–CH_3_ bonds within synthetically conjugated methyl groups. Additional CH_3_ bending was observed at 1455 cm^−1^. Ether bonds (C–O–C) originating from phenolic groups were identifiable at 1026, 1152, 1195, and 1287 cm^−1^. The presence of TMS could not be identified in the liposomes due to the absence of corresponding peaks. The individual components of the liposomal structure, 1,2-distearoyl-sn-glycero-3-phosphocholine and 1,2-distearoyl-sn-glycero-3-phosphoethanolamine-*N*-[methoxy(polyethylene glycol)-2000] ammonium salt were identifiable with peaks at 2950–2850 and 1730 cm^−1^, corresponding with the presence of alkane (C–H) and carbonyl ester groups (C=O). The broad peak observed at 3500 cm^−1^ was a result of the OH groups found within fatty acids (cholesterol). Additional vibrations corresponding with a C–C bond at 1248 cm^−1^ and a C–O bond from the phenolic group at 1154 cm^−1^ were identified within the spectrum (Figure 1C,D).

### 2.2. TMS-Loaded Liposomes Maintained Cell Viability In Vitro

**Figure 2:**
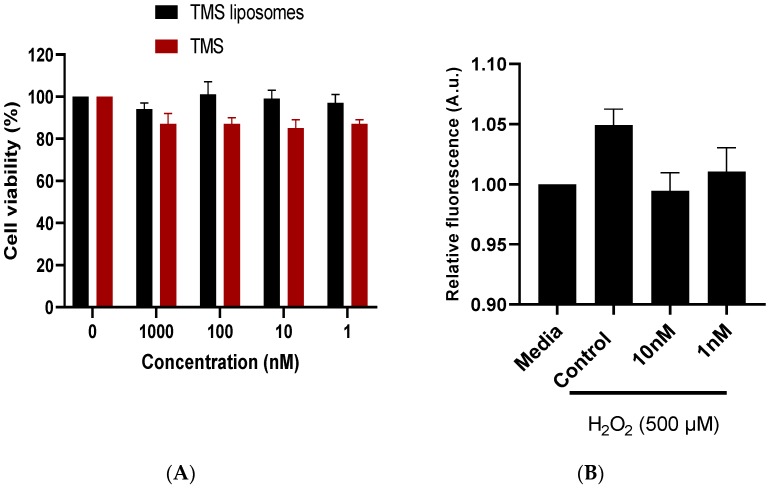
Effects of tetramethoxystilbene (TMS) and TMS-loaded liposomes on the viability and mitochondrial superoxide generation in human coronary artery endothelial cells (HCAECs). (**A**) Alamar blue cell viability assay performed in a 96 well plate following administration of cumulative doses of TMS and TMS-loaded liposomes for 24 h; (**B**) mitochondrial superoxide generation in HCAECs measured using MitoSOX Red reagent. Assays performed in a 96 well plate with cells treated with TMS-loaded liposomes for 24 h, followed by hydrogen peroxide (H_2_O_2_) 500 μM exposure. Untreated cells received media alone. Data are presented as mean ± standard error of mean, n = 3.

Human coronary artery endothelial cell (HCAEC) viability did not differ significantly between control cells and cells incubated with TMS or TMS-loaded liposomes for 24 h (Figure 2A). Drug encapsulation within liposomes has previously been shown to provide greater therapeutic efficacy with low cytotoxicity levels in human retinal endothelial cells [14]. In order to replicate the elevated superoxide levels observed in the vasculature during oxidative stress, HCAECs were stimulated with hydrogen peroxide (H_2_O_2_) [15]. A reduction in fluorescence intensity, and therefore mitochondrial superoxide production, was observed following incubation with TMS-loaded liposomes (Figure 2B).

### 2.3. TMS-Loaded Liposomes Restored Endothelium-Dependent Dilation via Nitric Oxide

**Figure 3:**
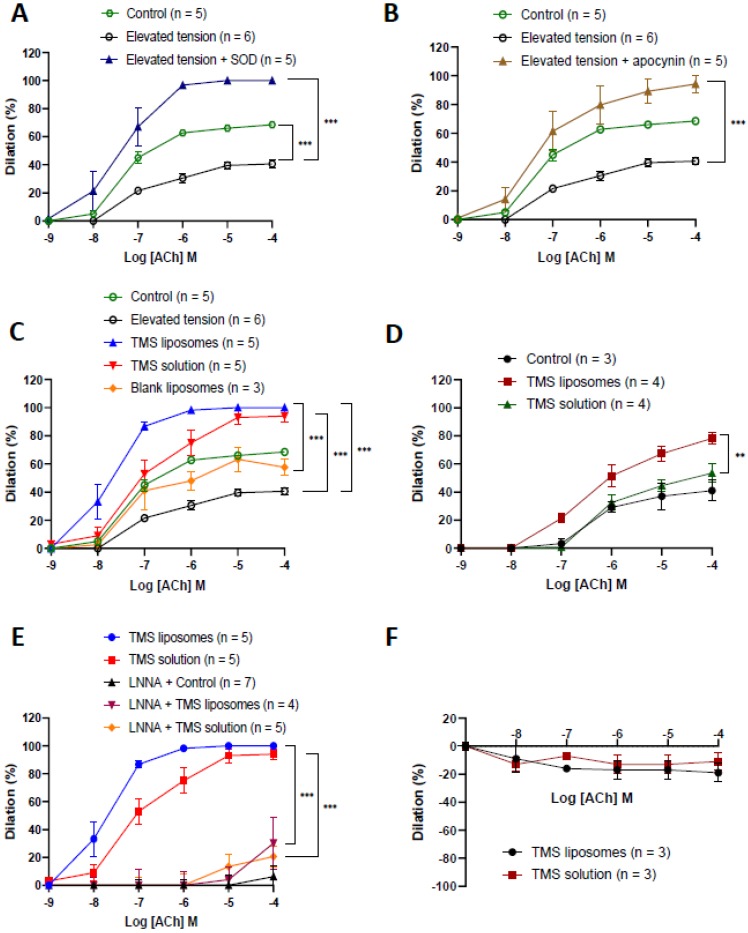
The effect of tetramethoxystilbene (TMS) and TMS-loaded liposomes on the vascular responses of isolated aortic vessels. Endothelium-dependent acetylcholine (ACh) responses in control vessels exposed to standard tension (control—2 g tension), following acute tension elevation (4 g), acute tension elevation + superoxide dismutase (SOD) (**A**) and tension elevation + apocynin (**B**) in phenylephrine pre-constricted aortic vessels ex vivo. The influence of tetramethoxystilbene (TMS) and TMS-loaded liposomes (**C**), 4 h after exposure to TMS and TMS-loaded liposomes (sustained responses) (**D**), in the presence of the inhibitor Nω—nitro-l-arginine (L-NNA) (**E**) and after endothelial denudation (**F**) on endothelium-dependent ACh responses following acute tension elevation in phenylephrine pre-constricted aortic vessels ex vivo. ** = *p* < 0.01, *** = *p* < 0.001. Error bars = standard error.

All vessels constricted in response to high potassium (KPSS, 60 mM) and phenylephrine (Phe) (10^−5^ M) solution. Acute tension elevation significantly attenuated endothelium-dependent dilation to acetylcholine (ACh), which was restored following co-incubation with superoxide dismutase (Figure 3A) or the NADPH oxidase inhibitor apocynin (Figure 3B). This reduction in dilation following tension elevation was significantly potentiated after incubation with TMS or TMS-loaded liposomes. Incubation with blank liposomes did not alter ACh dilator responses (Figure 3C). The potentiated dilator effects were maintained for 4 h after exposure to TMS-loaded liposomes, but not TMS solution (Figure 3D).

There was a significant reduction in dilator responses following inhibition of nitric oxide synthesis by L-NNA (Figure 3E). Vessels did not dilate in response to ACh following acute tension elevation and incubation with TMS or TMS-loaded liposomes after endothelial denudation (Figure 3F). TMS and TMS-loaded liposomes had no effect on endothelial-independent (SNP) dilation (data not shown).

### 2.4. TMS-Loaded Liposomes Demonstrated Better Efficacy of ACh-Induced Dilation than TMS Solution

**Figure 4:**
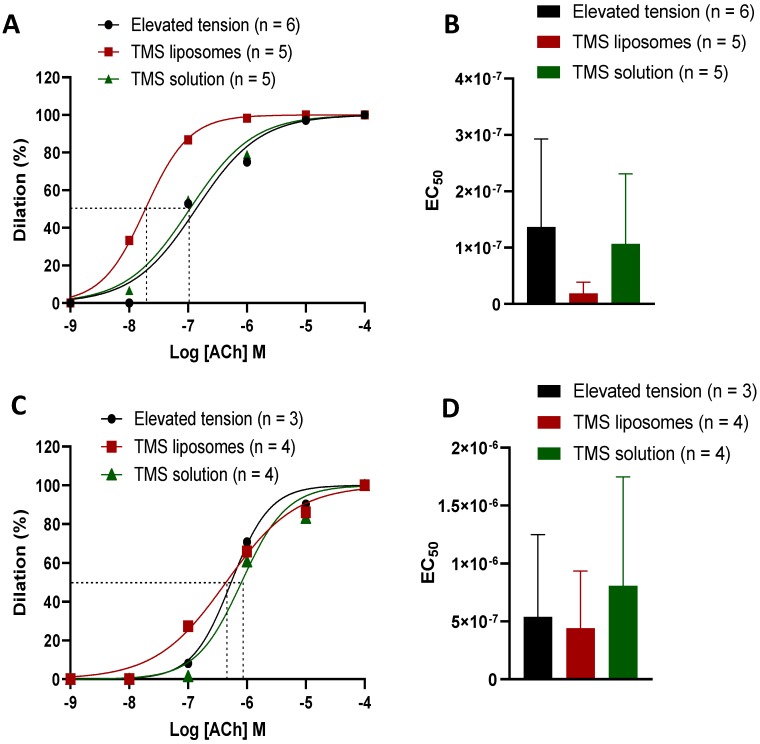
The EC_50_ shows the initial (**A**,**B**) and sustained (**C**,**D**) effects of tetramethoxystilbene (TMS)-loaded liposomes in comparison to TMS solution on dilator responses. Error bars = standard error.

TMS-loaded liposomes, but not TMS solution, induced a leftward shift in the ACh-sensitivity curve of the dilator response (*p* ≤ 0.0001; Figure 4A,B). When dilator responses were examined 4 h after exposure to TMS/TMS-loaded liposomes (sustained responses), a rightward shift was observed with TMS solution, but not after exposure to TMS-loaded liposomes, demonstrating that the TMS-loaded liposomes sustained some of their effects (*p* ≤ 0.0001; Figure 4C,D).

## 3. Discussion

We demonstrated that TMS, loaded within liposomes, can restore the reduced aortic vasodilator response induced by a high oxidative environment by potentiating the NO pathway. We produced TMS-loaded liposomes for direct uptake into the vasculature. These were characterized and dye loading was confirmed using a range of chemical techniques. Due to the limited content that can be encapsulated within liposomes, a substantial absorbance peak was not expected and may, therefore, have been obscured by more prominent absorption peaks, as seen at 207 nm. This intense absorption towards the lower detection range has previously been observed in liposome samples and has been attributed to the presence of cholesterol. Cholesterol and other well-known oxysterols have been shown to absorb light at approximately 195 nm (vacuum–UV region). However, light scattering results in the *λ* max shifting towards a longer wavelength, producing an effect commonly referred to as false energy. This results in a spectral “redshift”, with spectra then appearing in the 207 nm region [16]. Using FTIR, we identified several peaks which corresponded with the parent compound RV, confirming its derived nature. FTIR also confirmed the presence of constituent compounds used to synthesise the liposomes; however, the spectrographic profile for TMS was not distinguishable. Similar observations have been reported previously by several research groups, where nanoparticles are capable of obscuring the absorption peaks of encapsulated drugs, showing only the predominant parent compounds [17]. In the context of polyphenolic compounds, the presence of OH groups is commonplace and would be expected at around 3500 cm^−1^. Despite being synthesized from a polyphenolic parent compound, it is crucial to note that in TMS, OH groups have been substituted for synthetically conjugated methyl groups, and as a result valance vibration bands at 3500 cm^−1^ would not be expected. This, in addition to the presence of the same broad peak on blank liposomes, was suggestive that the vibration observed was solely from the lipid components. The distribution and intensity of absorption (% transmission) bands (blank vs. TMS-loaded liposome) are a direct reflection of the range and quantity of chemical bonds present within a given sample. In some instances, this may reflect an increase or decrease in band intensity as a result of sample modification and altered chemical composition. In this case, however, differences in intensity observed between samples can be attributed to different dilution ratios of stock samples.

Using isolated HCAECs in culture, we demonstrated that TMS-loaded liposomes can maintain cell viability. When the cells were exposed to H_2_O_2_, mitochondrial superoxide generation was reduced following a 24 h incubation with TMS-loaded liposomes. The lack of significance in reduction suggests that TMS-loaded liposomes may have quenched additional sources of ROS, including cytosolic superoxide, due to NADPH oxidase stimulation and NOS uncoupling [15]. Furthermore, TMS-loaded liposomes may also have influenced the upregulation of antioxidant enzymes and eNOS expression in a similar way to resveratrol [18,19]. To demonstrate the vasodilatory potential of the TMS-loaded liposomes, we used aortic vessels, exposed to elevated tension, to generate an oxidative environment. The upregulation of ROS sources, particularly NADPH oxidase, decreases SOD activity and causes eNOS uncoupling [2]. Vessel incubation with the NADPH oxidase inhibitor apocynin significantly potentiated dilator responses following tension elevation. This is consistent with previous findings that NADPH oxidase is the major ROS generator in the vasculature [20]. Apocynin blocks the translocation of p47phox to the membrane, hence preventing the release of NADPH-oxidase-derived superoxide [21].

Vessel incubation with TMS/TMS-loaded liposomes restored endothelium-dependent dilator responses following tension elevation even beyond the maximal dilation observed in control vessels. TMS has been documented to reduce the increase in NADPH-oxidase-derived ROS in aortic, mesenteric, and renal arteries of spontaneously hypertensive rats (SHR), primarily via inhibition of CYP1B1 activity. CYP1B1 has been reported to contribute to ED in SHR, with TMS reversing this effect, as indicated by improvement in vasodilation in response to ACh [7]. It appears that ED in SHR and DOCA hypertensive models occurs largely as a result of elevated ROS generation, resulting from arachidonic acid (AA) metabolites such as 20-HETE, which is produced by CYP1B1. This subsequently elevates NADPH oxidase activity and ROS levels, resulting in the activation of signaling molecules including ERK1/2, p38 MAPK, and c-Src [22]. This was prevented by TMS treatment [8]. The potentiation in dilator responses demonstrated by TMS could be attributable to increased eNOS phosphorylation, reduced NADPH-oxidase-derived ROS and eNOS uncoupling via CYP1B1 inhibition, direct quenching of ROS, and the activation of SIRT1 and/or AMPK pathway, leading to elevated NO bioavailability. Inhibition of NO synthesis using L-NNA significantly attenuated the vasodilation restored by TMS/TMS-liposomes, suggesting that its mechanism of action is mainly mediated via the NO pathway, which plays a major role in the maintenance of basal vasomotor tone in conduit vessels under both normal and hypertensive conditions [23]. The dilator potential of TMS/TMS–liposomes was lost in denuded vessels, further suggesting that the potentiated dilation is mainly mediated via endothelial dependent mechanisms.

TMS and TMS-loaded liposomes resulted in an acute improvement in the dilator response capacity following tension elevation. In addition, TMS-loaded liposomes, but not TMS solution alone, increased sensitivity to ACh. We also demonstrated that the enhanced endothelial-dependent dilator response to ACh was sustained 4 h after exposure to TMS-loaded liposomes, but not to TMS solution. Previous studies using isolated endothelial cells [24,25,26] and uterine vasculature [27] have documented receptor-mediated endocytosis to be the main mechanism of liposomal uptake. The physiological effects we observed were likely due to increased cellular uptake of liposomes, enhanced solubility, and sustained release of encapsulated TMS [12], as compared to the rapid depletion/washout of TMS solution in the experimental setup.

## 4. Materials and Methods

### 4.1. Chemicals, Reagents, and Materials

2,3′,4,5′-Tetramethoxystilbene (TMS) and all reagents were purchased from Sigma-Aldrich, UK. Physiological salt solution (PSS) had the following composition [mM]: 119 NaCl, 4.7 KCl, 1.2 MgSO_4_7H_2_O, de-ionized (dH_2_O), 25 NAHCO_3_, 1.17 KHPO_4_, 0.03 K_2_EDTA, 5.5 glucose, 1.6 CaCl_2_H_2_O; pH 7.4.

### 4.2. Liposome Synthesis and Characterisation

Liposomes were synthesized using the thin lipid film process as previously described [27,28]. Briefly, the constituent lipids 1,2-distearoyl-*sn*-glycero-3-phosphocholine (DSPC; 32.5 mM, Avanti Polar Lipids, Alabaster, AL, USA), 1,2-distearoyl-*sn*-glycero-3-phosphoethanolamine-*N*-[methoxy(polyethylene glycol)-2000] ammonium salt (DSPE-PEG(200); 1.875 mM, Avanti Polar Lipids) and cholesterol (15 mM, Sigma-Aldrich, St. Louis, MO, USA) were dissolved in 5 mL of chloroform in a round-bottomed flask. Subsequent rotary evaporation (40 °C, 270 mbar) removed excess chloroform and the lipid film was placed in a vacuum oven overnight (25 °C, 0 mbar). The thin film was rehydrated with 1 mL of PBS, vortexed for 10 min, heated to 55 °C, and vortexed for 5 min at 1 h intervals for a minimum of 4 h. The suspension containing large multilamellar vesicles was then extruded 11 times using a 1 mL Mini-Extruder (Avanti Polar Lipids) through a 200 nm polycarbonate membrane to produce unilamellar liposomes ~200 nm in diameter. To remove impurities, dialysis against PBS (8 × 500 mL; 24 h) in a Slide-A-Lyzer cassette with a molecular weight cutoff of 3.5 kD (Thermo Fisher Scientific, Waltham, MA, USA) was performed. Liposomes were then stored at 4 °C until use. The size distribution and polydispersity index of liposomes were measured by dynamic light scattering (DLS; 25 °C; scattering angle of 173° Zetasizer Nano ZS, Model ZEN3600, Malvern Instruments, Malvern, UK). For TMS-loaded liposomes, the lipid film was rehydrated with 1 mL TMS (2 mM in PBS) heated to 55 °C and extruded/dialysed as above, to give a final encapsulated concentration of approximately 1 mM TMS. For dual-loaded TMS/5(6)-carboxyfluorescein liposomes, a 1 mL solution of TMS (2 mM) containing 5(6)-carboxyfluorescein (5.3 mM) was used to rehydrate the lipid film to give final concentrations of 1 mM and 2.65 mM, respectively. A UV-Vis spectrophotometer (Jenway 7305, Cole-Palmer, Vernon Hills, IL, USA) and fluorescence spectrometer (F-7000, Hitachi, Tokyo, Japan) were used to determine absorption and fluorescence spectra, respectively. The structure of liposomes was assessed using FTIR (Nicolet 380 FT-IR Spectrometer, Thermo Scientific).

### 4.3. Cell Culture Studies

HCAECs were purchased from PromoCell (Heidelberg, Germany). Cells were grown in Endothelial Cell Growth Medium (MV2) supplemented with growth medium MV2 supplement pack, 100 μL/mL penicillin and 100 μL/mL streptomycin. Cells were maintained at 37 °C with 4% CO_2_ in a humidified incubator, and subcultured 1:3 after reaching 80% confluency. The viability of HCAECs was determined using the fluorometric Alamar Blue Cell Viability assay. HCAECs were seeded in 96 well plates and exposed to increasing doses of TMS solution and TMS–liposomes (1 nM–1 µM) for 24 h. Fluorescence was measured at ex. 570/em. 590 nm using a BioTek Synergy HT microplate reader. The generation of intracellular superoxide was quantified using MitoSOX Red Mitochondrial Superoxide Indicator (Thermo, UK). Briefly, HCAECs were cultured on 96 well plates at a density of 1 × 10^4^ cells/well for 24 h. Cells were then incubated with TMS-loaded liposomes (10 and 1 nM) for 24 h, washed, then incubated with fresh medium in the presence of 500 μM H_2_O_2_ for 30 min. Cells were incubated in the dark at 37 °C with MitoSOX for 10 min. Fluorescence was measured at ex. 510/em. 580 nm (BioTek Synergy HT microplate reader).

### 4.4. Vascular Function Studies

Aortic vessels were excised from male Wistar rats (150–250 g) euthanized by stunning and cervical dislocation, following Institutional guidelines and in accordance with the “Animals Scientific procedures act 1986”. The heart was isolated and kept in oxygenated PSS (95% O_2_: 5% CO_2_; 4 °C) in a dissection dish. The aortic vessel was gently pinned down at both ends and the underlying adipose tissue was removed using dissection scissors and forceps under a binocular dissection microscope with a fibre optic light source (Olympus SZ61). The vessel was then cut into 3 mm rings and mounted between two fine steel wires in a calibrated organ-bath system, immersed in oxygenated PSS at 35 °C, and then placed under 2 g tension using a Harvard isometric strain transducer and tension was recorded using Labchart 8 (Power lab, AD Instruments, Oxford, UK), as previously described [29]. The viability of blood vessels was examined by ascertaining initial responses to high potassium solution (KPSS, 60 mM). Vessels that recorded constrictor responses of <1.0 g tension were not considered viable and were excluded from the study. The influence of TMS/TMS-loaded liposomes on blood vessel function was determined in response to vasodilator agonists including acetylcholine (ACh) (10^−9^ M–10^−4^ M), sodium nitroprusside (SNP) (100 nM), and the vasoconstrictor phenylephrine (Phe) (10^−5^ M).

Endothelial-dependent (ACh) and-independent (SNP) responses were examined following acute tension elevation (4 g, 1 h) in the presence/absence of TMS/TMS-loaded liposomes. In order to establish the minimal dose of TMS required to achieve maximal dilation, cumulative doses of TMS (1 pM–10 mM) were added to vessels pre-constricted with Phe. In order to assess the dilator effect of superoxide dismutase (SOD) and TMS/TMS-loaded liposomes, aortic rings were initially placed under elevated tension (4 g, 1 h), followed by a 30 min incubation with SOD (300 U/mL) or TMS/TMS-loaded liposomes (1 nM). Constrictor and initial dilator responses were investigated and recorded. To evaluate the dilator component involved in the action of TMS/TMS-loaded liposomes, inhibition studies were performed. L-NNA (NO synthesis inhibitor; 100 μM) was used to evaluate the influence of NO following acute tension elevation with and without incubation with TMS/TMS-loaded liposomes. To assess the contribution of NADPH oxidase to the dilator function of TMS/TMS-loaded liposomes, apocynin (0.3 mM, 30 min) was used to block NADPH oxidase activity. In a separate set of experiments, vascular responses were assessed 4 h after exposure to TMS/TMS-loaded liposomes (sustained responses). Endothelial denudation was used to assess the endothelial-dependent dilator component of TMS/TMS-loaded liposomes. To investigate any effects of the liposomes alone on vasodilation, aortic vessels were incubated with blank liposomes (drug-free) (1 nM) for 30 min after tension elevation. Constrictor and dilator responses to Phe and ACh were measured, respectively.

### 4.5. Statistical Analysis

All values are presented as mean ± standard error. Data were tested for normal distribution using Shapiro–Wilk test. Results were analysed using a two-way ANOVA followed by Tukey’s multiple comparisons post-test (IBM SPSS Statistics 25). Statistical significance was *p* < 0.05. * = *p* < 0.05, ** = *p* < 0.01, *** = *p* < 0.001. *p* > 0.05 was considered non-significant.

## 5. Conclusions

In the present study, isolated ex vivo aortic vessels exposed to elevated tension exhibited impaired dilator function. We demonstrated that TMS-loaded liposomes can restore the attenuated endothelial-dependent dilator responses following tension elevation, by reducing NADPH-oxidase-derived ROS and potentiating NO-mediated dilation. The restoration was sustained after exposure to TMS-loaded liposomes, but not TMS solution, suggesting that TMS-loaded liposomes may present a promising therapeutic strategy to improve vasodilator function in vascular disease.
